# Rouse and Zimm Short-Time Exponents When Subtracting the Solvent Contribution in Semidilute Polymeric Solutions

**DOI:** 10.1021/acs.macromol.5c00371

**Published:** 2025-07-23

**Authors:** Pablo Domínguez-García, Sylvia Jeney

**Affiliations:** † Dep. Física Interdisciplinar, Universidad Nacional de Educación a Distancia (UNED), Madrid 28040, Spain; ‡ Department of Physics, 27211 University of Fribourg, Fribourg CH-1700, Switzerland

## Abstract

Optical trapping interferometry measures the Brownian motion of an optically trapped microsphere inside a fluid, allowing access to the short-time/high-frequency power-law behavior appearing in the complex modulus of polymeric aqueous suspensions, which is characteristic of the individual dynamics of the macromolecules. The short-time behavior is shaped by the hydrodynamics and inertia effects because of the solvent, whose influence in the complex modulus we analyze through inertia-corrected expressions and by removing the contribution of the solvent. We detect that the classic Zimm and Rouse models in the high-frequency region reproduce the experimental results for aqueous suspensions of poly­(ethylene oxide) and of worm-like micelles in the semidilute regime when the water contribution is adequately subtracted. Our results reflect the incompleteness of the Zimm–Rouse model for including full hydrodynamic interactions but also reveal how the transition between different dynamic behaviors of the polymers in suspension depends on the network interconnection.

## Graphical abstract

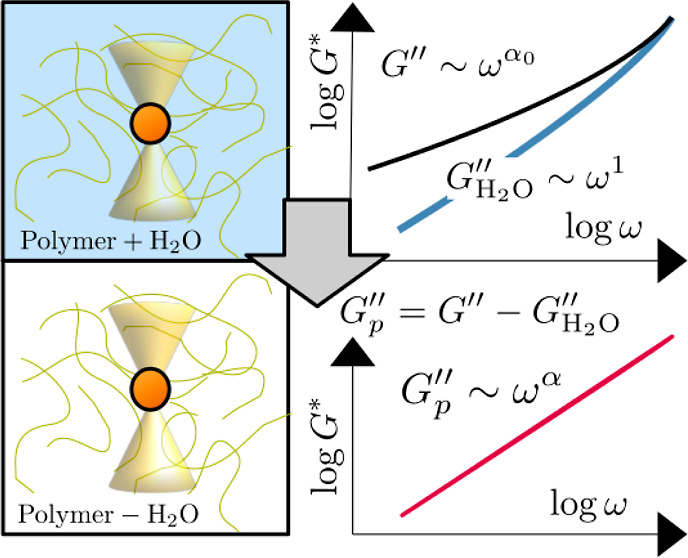

## Introduction

The Rouse–Zimm model^1,2^ is the well-known classic theoretical approach to reproduce the dynamical properties of flexible macromolecules. Basically, each molecule is considered to be formed by Brownian beads linked by spring forces, forming linear ideal (Gaussian) chains which are of the same length and whose end-to-end distance follows a Gaussian distribution.^3^ The main difference between Rouse and Zimm models is that the former neglects the interaction with the solvent, which is considered static, whereas the latter uses ideal chains with hydrodynamic interactions, and therefore, the motion of each bead affects the other ones through the flow generated in the solvent. However, in the simpler version of the Zimm model, the inertial effects of the beads and the solvent are also neglected, something that corresponds to the assumption of a small Reynolds number, allowing the simplification and linearization of the hydrodynamic Navier–Stokes equation.^4^

In spite of their limitations, these models allows us to obtain the response of different polymeric materials, such as melts or polymeric suspensions, to shear strain.^5^ This response is viscoelastic in nature, and it is characterized by the stress relaxation modulus, *G*_r_(*t*), whose Fourier transform returns the frequency-dependent complex shear modulus, G*­(ω). The real part of G*­(ω) gives the elastic response of the material to an oscillatory strain, *G*′(ω), whereas the imaginary part returns the loss modulus o viscous component, *G*″(ω). Time scales are particularly determinant in the description of the viscoelasticity of soft materials, especially in terms of characteristic relaxation times and the application of single-relaxation-time Maxwell models.^6^ In the context of Doi–Edwards theory,^5^ and for a sufficiently short time scale, τ_e_, polymer dynamics can be described by reptation motion, i.e., their diffusion is constrained by an imaginary tube composed by the surrounding polymers. The tube model provides then a stress relaxation modulus, which follows a temporal power-law behavior^7^ in the form $G_{r}\left(t\right)≅G_{t⩽\tau_{e}}{\left(\tau_{e}/t\right)}^{\alpha }$ , with *t* ⩽ τ_e_, and where the values of $G_{t⩽\tau_{e}}$ and α depend on the characteristics of the polymers and the solvent. However, at very short time scales, the influence of the solvent cannot be ignored.^8^ Inertia and hydrodynamic memory have been included in relation to the Rouse model^9^ and when modeling the Brownian motion of polymers in suspensions,^4,10^ and they have been experimentally observed in nanoconfined DNA.^11^

The effect of the solvent also has an influence when using Brownian microprobes to experimentally explore the characteristics of their surrounding fluid.^12^ The analysis of this Brownian motion to extract the micromechanical properties of the object’s surroundings composes a research field called microrheology,^13,14^ which has been systematically employed in the last three decades in the study of biophysics and soft matter.^15–18^ If the particle’s Brownian jumps are measured on the scale of the microsecond or lower, the ballistic region appears and the hydrodynamic memory is no longer negligible.^19^ In that experimental situation, an alternative expression is needed for calculating the complex modulus of the viscoelastic material, which includes inertia effects from the bead and the surrounding medium.^20,21^

In this work, we use optical trapping interferometry, an experimental technique which allows us to detect the Brownian motion of the probe in the microsecond range with nanometer accuracy.^22,23^ Using the data obtained from this experimental technique, we explore the power-law exponents, α, which appear in the complex modulus at high frequencies in typical viscoelastic fluids,^24,25^ such as water solutions of worm-like micelles and poly­(ethylene) oxide. The values of these power-law exponents are characteristic of the individual behavior of the macromolecules in the material.^26^ In order to generate a non-Newtonian response, the value of the polymer concentration, *c*, in the liquid needs to be on the order of or above the polymer overlap concentration, c*. In that case, the solution is called semidilute because the overlapped polymer occupies a very low volume fraction of the fluid, with most of the volume being formed by the solvent. Physical properties are dominated by the overlapping of the molecules, and when the polymer concentration is higher than the overlap concentration, the solution forms a transient mesh with contact between the polymer coils. Therefore, the ratio of the polymer concentration to the overlap concentration drives the entanglement of the polymer structures and the change from dilute to semidilute and to concentrated regimes (*c* ≫ c*).^27^ If the nondilute regime is reached, the Rouse model returns an exponent of 1/2 at high frequencies, which is the classic result for flexible chains in the melt. Because of its inclusion of hydrodynamic interactions, the Zimm theory is more appropriate for modeling polymeric solutions, providing α = 2/3, although, for a good solvent, the exponent should be 5/9.^5^ If the behavior of the polymers composing the fluid is semiflexible, a value of α = 3/4 at high frequencies is obtained,^28^ although the transition from the flexible to the semiflexible regime is not clear.^25^

We investigate here the power-law exponents in viscoelastic solutions through *G*″(ω) ∼ ω^α^ at frequencies between 10 kHz and 1 MHz, a high-frequency region where the complex modulus, G*­(ω), has a strong dependence on frequency and the loss modulus dominates over the elastic modulus.^29^ When using a periodic strain, *G*″(ω) determines the energy dissipation, which depends on both solute and solvent.^30^ Here, we assume that the stress tensor, which defines the linear viscoelasticity of a semidilute suspension of polymers, is composed of two parts: the viscous stress and the elastic stress.^5^ According to that, we experimentally evaluate the influence of the solvent by subtracting the loss modulus of water’s contribution to the values of the loss modulus obtained for different viscoelastic fluids.^31^

In this work, we observe that classical Rouse and Zimm theories reproduce some of the experimental curves of the loss modulus when the water contribution to the loss modulus is subtracted. Next, we explore the dependence of the obtained power-law exponents on the ratio of the polymer concentration to the overlap concentration, where we observe a notable difference between the data obtained from the complex modulus with or without removal of the solvent contribution. Our analysis is based on the application of standard theoretical expressions for the complex modulus of viscoelastic solutions at high frequencies, models that we detail hereunder for the sake of completeness.

### Expressions for the Complex Modulus

In this section, we detail the theoretical expressions for the complex modulus through the classic Rouse–Zimm model and the theory for semiflexible polymers. After that, we describe the standard methodologies used in microrheology for calculating the complex modulus, including the inertia-corrected expression. In Rouse–Zimm (RZ) theory, the complex moduli are expressed in the following form

1 $$G^{\prime }\left(\omega \right)=\frac{cRT}{M}\sum_{p=1}^{\infty }\frac{\omega^{2}\tau_{p}^{2}}{1+\omega^{2}\tau_{p}^{2}}$$

2 $$G^{\prime \prime }\left(\omega \right)=\omega \eta_{s}+\frac{cRT}{M}\sum_{p=1}^{\infty }\frac{\omega \tau_{p}}{1+\omega^{2}\tau_{p}^{2}}$$

where *c* is the polymer concentration in g/mL,^32^ *M* is the molecular weight, and η_s_ is the viscosity of the solvent. The relaxation times, τ_*p*_, where *p* = 1, 2, 3, ... is the mode number of motions of the polymer chains, are

3 $$\tau_{p}=A_{p}\frac{cRT}{M}\left(\eta -\eta_{s}\right)$$

where η is the steady-state viscosity of the solution, and *A*_*p*_ is different for Rouse and Zimm theories. In the former, *A*_*p*_ = 6/(π^2^ *p* ^2^), whereas in the latter, *A*_*p*_ = 1.71/λ_*p*_′, where λ_*p*_′ are the eigenvalues from the solution of the equation of motion of the polymer chain. The first values are λ_*p*_′ = 4.04, 12.79, 24.2,... and subsequent eigenvalues can be calculated using an approximate expression.^33^

Equations 1 and 2 are similar to those of the Maxwell fluid but include a spectrum of relaxation times. Only a few sets of complex fluids, such as solutions of telechelic polymers or micelles in specific conditions,^34^ follow the one-relaxation-time Maxwell model at low and intermediate frequencies. In viscoelastic systems like solutions of micelles, the stress relaxes first by Rouse–Zimm modes and then by the internal bending modes of Kuhn segments when the frequency increases.^28^ Then, the power-law exponent changes to a characteristic 3/4 value with the following expression for the complex modulus

4 $$G^{*}\left(\omega \right)=i\omega \eta_{s}+\frac{1}{15}\rho_{m}\kappa l_{p}{\left(\frac{-2i\zeta }{\kappa }\right)}^{3/4}\omega^{3/4}$$

where η_s_ is the solvent viscosity, ρ_m_ is the polymer concentration in length per unit volume, κ is the bending modulus, *l*_p_ is the persistence length, and ζ is the lateral drag coefficient. These quantities can be deduced using the standard theory of polymers^5^ and the experimental results for the complex moduli. The fit of eq 4 to the data obtained in our particular experimental system has been verified for solutions of worm-like micelles^35^ and myosin solutions.^36^

In optical trapping interferometry, we use single tracers of radius *a* immersed in a fluid of density ρ. By means of one-particle microrheology, the results may depend on the length scales of the system,^37^ as opposed to bulk rheology^38^ or two-point microrheology.^39^ The standard methodology for obtaining the complex modulus from the motion of the tracer is the use of the Generalized Stokes–Einstein relation (GSER)^15^

5 $$G_{GSER}^{*}\left(\omega \right)=\frac{dk_{B}T}{3\pi ai\omega \overset{®}{MSD}}$$

where MSD is the mean squared displacement MSD (*t*) ≡ ⟨Δ*r* ^2^(*t*)⟩ = ⟨(*r*(*t*) – *r*(0))^2^⟩, *d* is the number of dimensions used for its calculation, and $\overset{®}{MSD}\equiv F\left\{\langle \Delta r^{2}\left(t\right)\rangle \right\}$ is its one-sided Fourier transform. The MSD is a quantity directly obtained from the detected position of the particle, while its Fourier transform is usually estimated using the Mason’s approximation.^40^ Alternative methodologies to eq 5 for estimating the complex modulus are, i.e., the classical Kramers–Kronig approach, which returns analogous values to the GSER with Mason’s approximation,^41^ or the straightforward expression by Evans et al.,^42^ which generates an excess of noise in our data.^43^

The GSER has a number of limitations: noninclusion of external forces, neglect of the coupling of the probe to the system by compressional modes,^31^ or the absence of inertial effects. By using the GSER, the external optical trap will appear in the experiments as a low-frequency component of the elastic modulus,^12^ although the contribution of linear and nonlinear optical traps can be subtracted by an alternative time-domain strategy.^44^ The compressional modes have been thoughtfully addressed in the context of two-particle microrheology.^45^ The contribution of the inertia on the complex modulus was accounted for by a concrete analytic expression, named as IGSER, under the condition of an incompressible medium (in three dimensions and following the original notation)^21,44^

$$G_{I}^{*}\left(\omega \right)=\frac{k_{B}T}{i\omega \pi a\overset{®}{MSD}}+\frac{m^{*}\omega^{2}}{6\pi a}+\frac{a^{2}\omega^{2}}{2}\times \left[\sqrt{\rho^{2}+\frac{2\rho }{3\pi a^{3}}\left(\frac{6k_{B}T}{\left({\left(i\omega \right)}^{3}\overset{®}{MSD}\right)}-m^{*}\right)}-\rho \right]$$

This equation can be directly related to eq 5, since the squared root term is equal to $6k_{B}T/\left({\left(i\omega \right)}^{3}\overset{®}{MSD}\right)=-\left(6\pi a/\omega^{2}\right)G_{GSER}^{*}\left(\omega \right)$ . Therefore, the inertia-corrected expression to be used along with eq 5 is^35^

6 $$G_{I}^{*}\left(\omega \right)=G_{GSER}^{*}\left(\omega \right)+\frac{m^{*}\omega^{2}}{6\pi a}+\frac{a^{2}\omega^{2}}{2}\times \left[\sqrt{\rho^{2}-\frac{2\rho }{3\pi a^{3}}\left(\frac{6\pi a}{\omega^{2}}G_{GSER}^{*}\left(\omega \right)+m^{*}\right)}-\rho \right]$$

where m* = *m*_part._ + 2πρ*a* ^3^/3 is the effective mass of the particle.^46^ By experimentally obtaining *G*_GSER_*­(ω) and then employing the IGSER expression, eq 6, we will deduce a theoretical estimation of the complex modulus without the influence of the effect of bead and medium inertia.

## Experimental Methodology

In our experimental setup, a Brownian probe is embedded in a complex fluid as a detector of its local environment in order to perform quantitative microrheology. In the optical trapping interferometry (OTI) technique, optical trapping^47^ of the Brownian particle is combined with interferometry for the detection of its position.^48^ The details of the experimental technique have been thoroughly described elsewhere,^22,23,49^ but the issue of the calibration of the interferometry detector, i.e., how to convert the units of the measured position of the probe from volts to meters, requires additional discussion. When the particle is located inside of a Newtonian fluid, the calibration can be done by fitting the experimental data to the analytical solution of Langevin equations on this physical situation.^23,46,50^ If the probe is immersed in a non-Newtonian fluid, different experimental approaches can be carried out,^51–54^ one of them is the method of the double flow chamber.^35^ This methodology consists of dividing the cell into two parts: one contains water, and the other contains the viscoelastic fluid. Under these conditions, a Brownian probe in the water chamber is located in the middle of the cell using the optical tweezers, and then a calibration experiment is performed. In the same experimental conditions, we subsequently measure the position of a trapped particle in the part of the cell that holds the viscoelastic solution. The data obtained from the Newtonian fluid allows us to calculate the volts-to-meter conversion factor by applying our own developed software.^55,56^ Then, we assume that this conversion factor is also valid for the measurements deduced from the second cell, something we have already confirmed by comparing the results obtained from diffusing wave spectroscopy (DWS) and OTI in viscoelastic fluids.^35^ In our experimental setup, the quartz cell containing the liquids has *xy* dimensions of ∼2 × 0.5 cm and a height of ∼100 μ m. In both liquids, we have previously added 1 μL of a water suspension with probe beads at a volume fraction of the particle in the solution equal to 10^–3^ wt %, assuring a separation between particles of at least 15 μm.

These probes are monodisperse spherical microbeads composed of chemically inert melamine resin with a density of ρ = 1570 kg/m^3^ (Microparticles, GmbH). The radius of the microbeads initially used in these experiments (performed at *T* = 294 K) is *a* = 0.94 μm, but we will also use *a* = 1.47 μm in some of the viscoelastic fluids for detecting variations because of inertial effects. The complex modulus, G*­(ω) = *G*′(ω) + *iG*″(ω), where *G*′ is the storage or elastic modulus and *G*″ is the loss modulus, can be experimentally deduced by using eq 5, and so is the mean square displacement (MSD), which is directly calculated from the position of the probe tracers, i.e., MSD­(*t*) = ⟨(*x*(*t*) – *x*(0))^2^⟩, where *x*(*t*) is the one-dimensional position of the particle (*d* = 1 in eq 5). The points of the experimental MSDs are divided into blocks formed by equally distributed intervals in the abscissa on a logarithmic scale. This blocking methodology allows us to calculate an error value for every averaged point inside the block.^57,58^ We block the data in ten bins per decade, allowing a good visualization of the data in a double-axis logarithmic scale.

In this work, we are interested in the short-time power-law behavior of viscoelastic solutions. Although the elastic and the loss modulus should behave similarly at high frequencies in this kind of fluids, *G*′(ω) suffers from limitations on this aspect in comparison with *G*″(ω). The first aspect to be mentioned is that the elastic component is affected by the potential generated by the focused laser beam on the probe, which is harmonic. The potential well is characterized by a spring constant *k* in its central region^59^ when nonlinearities^60^ or the influence of the limiting wall^61^ are negligible. The resulting force generates a component in the elastic modulus *G*_*k*_′ = *k*/6π*a*, and therefore, in these experiments, we only use the lowest value of the optical force available, *k* = 7 μN/m for resin probes with *a* = 0.94 μm (and *k* = 11 μN/m for *a* = 1.47 μm). Besides, in our system, *G*′(ω) suffers from a characteristic breakup, which limits the detection of this quantity at frequencies higher than 10 kHz. This breakup might be caused by several factors, like the sensitivity to artifacts^62,63^ or to numerical calculations at low values of *t* ^31,40^ (See the Supporting Information document for further details). Therefore, the viscous modulus is more appropriate than the storage modulus for studying the power-law behavior at high frequencies. In that frequency region, where the inertia effects appear, the influence exerted by the optical trap is minimal, and *G*″(ω) dominates the viscoelastic response by an order of magnitude.

### Materials

The viscoelastic fluids used here are aqueous solutions of poly­(ethylene oxide) (PEO) and worm-like micelle solutions.^64^ The steady-state viscosities, η_0_, of these fluids have been measured using a Rheometer MCR502 (Anton Paar, Austria). We explore an extensive range of viscoelastic PEO solutions by using molecular weights *M*_w_ = 231, 495, and 747 kDa and concentrations in the range of 0.5–15 mg/mL. PEO solutions contain classic polymers, i.e., flexible structures whose stress relaxation, above the overlap concentration and at intermediate time scales, is dominated by reptation. For these flexible polymers, the molecules diffuse inside an imaginary tube,^65^ and, at very short-times or high frequencies, hydrodynamic interactions are important.^66^ The overlap concentration, c*, for PEO solutions, is usually estimated^67^ by using the expression $c^{*}=M_{w}{\left(4/3\pi R_{g}^{3}N_{A}\right)}^{-1}$ , where *N*_A_ is the Avogadro number, *M*_w_ is the molecular weight, and *R*_g_(nm) = 0.0215*M*_w_^0.58^. Additionally, the mesh size of the polymeric network can be obtained^67^ by $\xi =R_{g}{\left(c^{*}/c\right)}^{0.75}$ , providing values of a few nanometers for every fluid studied here.

The worm-like micelle (WLM) solutions were prepared by standard protocols^68,69^ and are composed of surfactant cetylpyridinium chloride (CPy^+^ Cl^–^, Sigma-Aldrich, molecular weight 358.00 g/mol) and sodium salicylate (Na^+^ Sal^–^, Sigma-Aldrich, molecular weight 160.10 g/mol), with a molar ratio of [NaSal] = [CpyCl] = 100 mM and concentrations in the range 1–4 wt %. Typically, in this kind of micelle solutions, the molecular structures have diameters of 2–3 nm and contour lengths on the order of 0.1–1 μm.^70^ The mesh sizes are 10–30 nm, and therefore, our probe sizes are big enough for detecting the medium as a continuum, assuring the use of microrheology. For CPyCl/NaSal aqueous solutions, the overlap concentration is typically c* = 0.3 wt %.^70^ Therefore, the concentrations used in our experiments (1, 2, and 4 wt %) in the semidilute regime ensure *c* > c*.

WLMs generate structures in a permanent process of breaking and recombining. The reptation model was extended by Cates and Candau^64^ to include the particular dynamical behavior of these so-called “living polymers”. This breaking and reforming behavior makes their molecular weight distribution dependent on surfactant concentration. Unlike ordinary polymer solutions,^5,65^ like PEO solutions, where the molecular weight distribution is fixed, the reversibility of the self-assembly process of WLMs generates systems named “equilibrium polymers”, whose molecular weight distribution is not constant but in thermal equilibrium.

Regarding the micromechanical properties of these WLM solutions, the simultaneous processes of reptation and scission appear on the relaxation of stress when the polymeric structures are sheared. The characteristic time that defines the stress relaxation of the fluids containing WLMs is precisely the breaking/recombination time of these molecules. If this time is shorter than the reptation time, the breaking/recombination processes generate a Maxwellian behavior, i.e., a monoexponential stress relaxation.^7^ However, at high frequencies, the bending fluctuations of individual micelles generate a continuous spectrum of relaxation times. As a consequence, at sufficiently high frequencies, the micelle structures in solution compose a fluid formed by semiflexible polymers,^25^ providing the power-law form of the moduli as a result^30^ and following the mechanical behavior marked by eq 4. In summary, while classic PEO polymers generate quite uniform and stable flexible structures, living polymers generate more dynamic coils, which are very influenced by the polymer concentration and even by the force generated by the external optical trap.^12^

### Subtracting the Solvent

In summary, we investigate the shape of the curves of the loss modulus at high frequencies for measuring the power-law exponents for different viscoelastic fluids to explore the influence of the inertia and the hydrodynamics over the experimental values of these exponents. We obtain the complex modulus by means of the standard microrheological calculation by using the GSER, eq 5, and its inertia-corrected version, eq 6. When the viscous modulus is measured, a quantity is obtained which adds the contribution from polymer and buffer.^25^ The part of the solvent is usually extracted by subtracting ωη_s_ (see eqs 2 and 4), and it has been demonstrated that the solvent contribution has to be deducted to observe Zimm modes in the high-frequency power-law behavior of solutions of flexible polymers.^66^ Here, the influence of the solvent can be traced by subtracting the loss modulus obtained from the water measurements. This can be accomplished thanks to the previously described method of the double chamber, where we consecutively measure the motion of a probe in water and a viscoelastic fluid under the same experimental conditions. By taking into account the loss modulus from water, we define a loss modulus *G*_*p*_″ = *G*″ – *G*″(H_2_O), which allows us to investigate the role of the solvent in the effects of inertia and hydrodynamic interactions in the high-frequency dynamics of single polymers in suspension.

Figure 1 summarizes our procedure when subtracting the solvent contribution: in the left column, we draw a representation of the bead located inside the fluid by the optical trap, whereas in the right column, we plot a schematic curve of the loss modulus at high frequencies. In Figure 1a, we plot the situation of the viscoelastic fluid formed by water as solvent and a polymeric structure as solute. In that situation, if we calculate the complex modulus using the GSER, we obtain that *G*″(ω) follows a power-law behavior (exponent α_0_) with an upward deviation because of inertia effects at frequencies above 10 kHz. The case with only water is plotted in Figure 1b, where the power-law behavior is diffusive (α_0_ = 1) and the inertia deviations also appear when ω grows.

**Figure 1:**
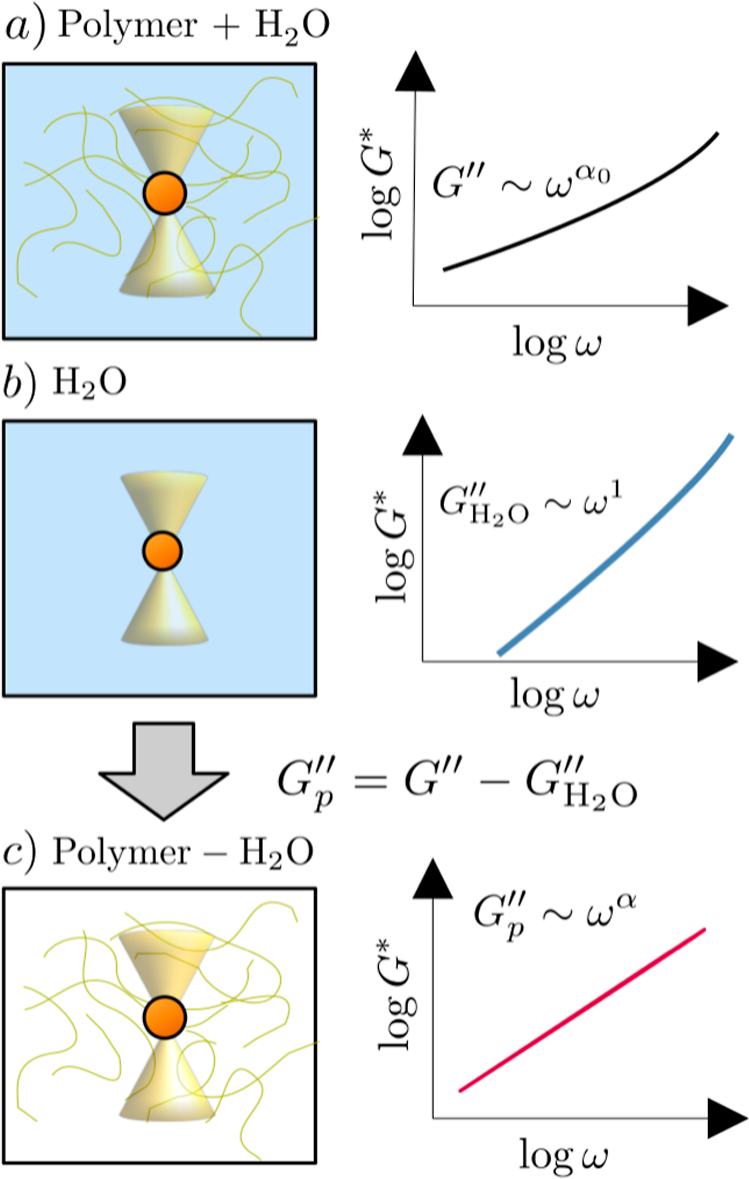
Schematic drawing of the experimental procedure of subtracting the influence of the solvent in the loss modulus, *G*″(ω), of the viscoelastic fluid. The left column is a representation of the microprobe (circles) located by the optical trap (yellow cones). In the column on the right, the expected curves of *G*″(ω) are plotted in the different cases: (a) polymer in water, (b) only water, and (c) viscoelastic fluid when the measurement of the loss modulus of the solvent is subtracted.

By subtracting the loss modulus of the solvent from the loss modulus of the viscoelastic fluid, we obtain a quantity *G*_*p*_″ which will follow a power-law behavior with an exponent α. This exponent is the result of subtracting the inertia and the hydrodynamic effects generated by the solvent, which are non-negligible at high frequency (ω ≳ 10^6^ Hz). Basically, these effects are composed of the inertia associated with the probe and the hydrodynamic resistance generated by the inertia of the fluid. Even at the condition of a low Reynolds’ number, the fluid is perturbed by the particle, which displaces the fluid because of the momentum received from the fluctuating molecules around it. Therefore, the so-called hydrodynamic memory^19^ causes the Brownian motion of the bead at short-time scales to depend on the nature of the surrounding medium. Moreover, due to the fluid, the bead suffers a resistance force composed of three components: the Stokes part, the inertia of the medium dragged around the bead, and the time-dependent Boussinesq–Basset friction force,^71,72^ which is defined by the penetration depth of the viscous and unsteady flow around the particle at all preceding times. This friction force has mixed contributions from the solvent and the polymer and acts like a pure viscous element at high frequencies.^21^

All of these components can be removed when calculating the complex modulus by using the IGSER expression, eq 6. We use that expression with the measurements of the polymer solutions and with the water measurements, and then we subtract one from the other to obtain *G*_*p*IGSER_″ = *G*_I_″ – *G*_I_″(H_2_O). Additionally, we calculate this quantity by using the data analyzed through the GSER, i.e., *G*_*p*GSER_″ = *G*_GSER_″ – *G*_GSER_″(H_2_O). Because the GSER does not remove the inertia effects, the comparison of the α exponents obtained by the analysis of these two *G*_*p*_″ allows a discussion about the relative weight of the bead inertia and the hydrodynamic memory in the dynamics of single polymers in suspension for the collection of viscoelastic fluids studied here.

## Results and Discussion

In these experiments, the optically trapped beads are immersed in two kinds of fluids: solutions of poly­(ethylene) oxide (PEO) and of worm-like micelles (WLM). For this reason, we separate the results section for the high-frequency micromechanical response of these fluids into two parts, one dedicated to classic polymers and the other to living polymers.

### Classic Polymers (PEO) Solutions

In Figure 2, we show three representative states of viscoelastic fluids formed by PEO solutions for different values of polymer molecular mass and concentration. In this figure, we plot the frequency-dependent viscous modulus obtained from the motion of optically trapped microbeads with *a* = 0.94 μm on a double logarithmic scale. Above the overlap concentration, mesh size ξ is the characteristic length for the polymeric network. Since the ξ values are approximately a few nanometers in PEO solutions with sufficiently large molecular weight,^12^ the size of the probe is large enough to detect the medium as a continuum.^14^ In the figure, the blue data correspond to values of the loss modulus, *G*″(ω), obtained from the motion of a trapped bead in water. The curves of the viscous modulus for the PEO solutions are calculated by four means: by using the GSER (*G*_GSER_″), by its inertia-corrected version IGSER (*G*_I_″), and by subtracting the loss modulus from water using the IGSER (*G*_*p*GSER_″) and using the GSER (*G*_*p*IGSER_″).

**Figure 2:**
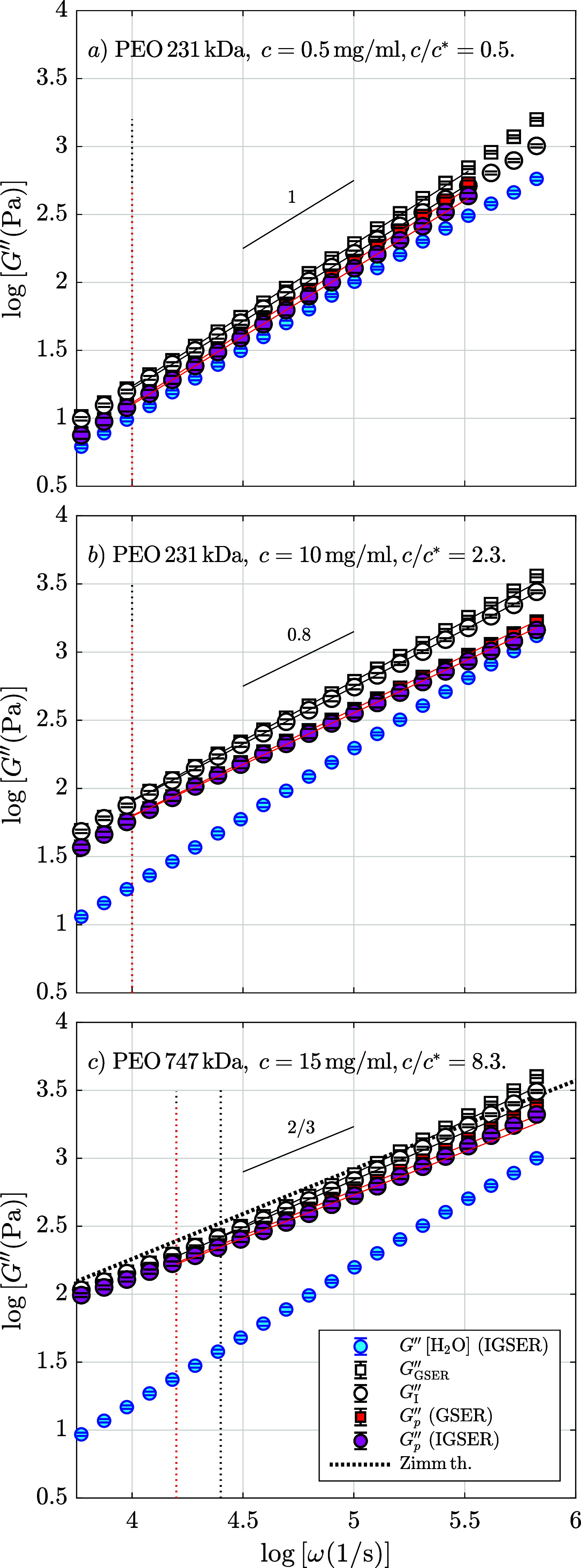
Loss modulus, *G*″(ω), obtained by optically trapped single-particle microrheology for three characteristic poly­(ethylene) oxide (PEO) solutions. The loss modulus is calculated by different methods: *G*_GSER_″, eq 5 (□); its inertia-corrected version *G*_I_″, eq 6 (○); and when subtracting water measurements using the GSER: *G*_*p*GSER_″ = *G*_GSER_″ – *G*_GSER_″(H_2_O) (■—red) and the IGSER: *G*_*p*IGSER_″ = *G*_IGSER_″ – *G*_IGSER_″(H_2_O) (●—rose). We plot the water measurements (●—blue), the curve from Zimm theory (.....), and linear regressions (—), where the vertical dotted lines indicate their lower boundaries. The data have been blocked in 10 bins per decade. Errors are plotted but are smaller than the point size. In (a) *c*/c* = 0.1, (b) *c*/c* = 2.5, and (c) *c*/c* = 8 with steady-state viscosity η_0_ = 35 mPa s.

Figure 2a shows a PEO solution with low molecular mass and low concentration as an example of the result obtained when the probe particle explores a dilute fluid, where *c* ≪ c*. In this situation, the macromolecules rarely overlap, and the particle does not detect a polymer network. As a result, the microrheology calculations return a Newtonian behavior^73^ with a power-law exponent α ≃ 1, similar to the pure solvent (in the figure, the blue points from water are nearby to those of the polymer solution). At low polymer concentration, the viscosity of the fluid will be proportional to the molecular weight, but above a critical value, the rate will increase because of the overlapping of the molecules, marking the beginning of the semidilute regime. In Figure 2b, we plot an intermediate situation between the dilute and semidilute regimes by using a PEO solution with *c*/c* = 2.5. Whereas the power-law exponent in the loss modulus for the Newtonian fluids is equal to 1, here this exponent value decreases, reaching a value of ∼0.8. In this panel, we can detect the difference in the loss modulus curves obtained from the PEO solution and from the solvent and also that *G*_*p*_″(ω) values deviate from *G*″(ω) and *G*_I_″(ω) ones. The third panel, Figure 2c, plots the viscous modulus for a ratio *c*/c* = 8. Here, the deviation from the Newtonian behavior of the solvent is noticeable. For this high-viscosity fluid, the power-law exponent is approximately equal to 2/3, the value from Zimm theory, which is more appropriate for classic polymeric solutions since it includes hydrodynamic interactions. The curve for the loss modulus obtained from Zimm theory (dotted points in Figure 2c) fits the experimental data.

We calculate the power-law values of the loss modulus curves obtained from a collection of PEO solutions at various molecular weights and polymer concentrations, including the three examples of Figure 2. The equally spaced data in double-axis logarithmic scale and the errors for the loss modulus values, estimated by error propagation using eqs 5 and 6, allow an adequate calculation of the α values.

These exponents are calculated by linear regressions, where we need to define criteria for the bottom and top limits. The upper boundary is set to be below the limit of our detection system^19^ at 10^5.7^ s^–1^. The lower limit is obtained by searching the last point where the residual corresponding to the top limit is larger than the value of reference (see the Supporting Information document). This bottom boundary is a value near 10^4^ s^–1^, which is related to the relaxation time of the properties of correlated polymeric structures.^66^ The result of the calculations is plotted in Figure 3, where α values are plotted versus the ratio *c*/c* of each suspension studied to show the dependence of the exponent on the grade of entanglement of the suspensions. When the suspension can be included in the unentangled regime, the exponent is equal to 1 and the suspension is purely Newtonian, but this behavior changes gradually when the ratio *c*/c* increases, approximating to the 2/3 Zimm value when *c* is near 1 order of magnitude higher than c*. As expected, the exponent from the noninertia-corrected modulus, *G*_GSER_″, does not reach the value 2/3 for our highest measured value of *c*/c*. The inertia-corrected curves improve this result, and the values of the power-law exponents are lower because the corrected loss modulus curves are straighter, partially removing the hydrodynamic effects that tend to move the curves upward at high frequencies (see Figure 1).

**Figure 3:**
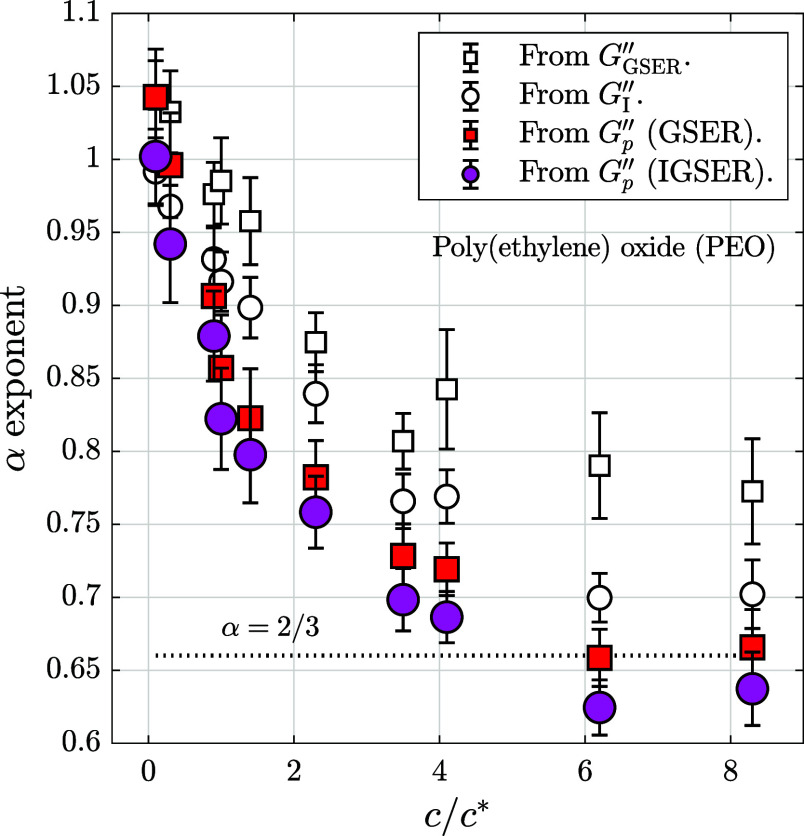
Experimental power-law exponents α in the high-frequency behavior of the loss modulus of poly­(ethylene) oxide (PEO) solutions in function of the ratio *c*/c*, where *c* is the polymer concentration and c* the overlap concentration. The values of α are obtained from linear regressions from *G*_GSER_″ (□), *G*_I_″ (○), *G*_*p*GSER_″ (■—red), and *G*_*p*IGSER_″ (●—rose).

Therefore, in Figure 3, we observe that both the GSER and IGSER exponent values do not match the exponent of Zimm theory, at least up until the higher values of the *c*/c* ratio measured here, but with the IGSER providing a much closer result to the expected theoretical value than the GSER, thanks to its inertia corrections. On the contrary, when subtracting the solvent using the GSER (*G*_*p*GSER_″), we obtain a similar descending curve with *c*/c*, showing a very good agreement at the last points with α = 2/3. However, this same methodology, but using the IGSER (*G*_*p*IGSER_″), provides α values slightly lower, although in the range of the error interval. The differences in the results for *G*_*p*_″ using these two methods suggest discrepancies between the assumptions about the polymer–solvent interactions in the Zimm theory and the inertial and hydrodynamic effects in these polymer solutions.

In the Zimm theory, the force of each bead is composed of the force due to their Brownian motion, the Hookean forces between two adjacent chains, and the hydrodynamic drag force by the solvent.^30^ Then, in that model, the interactions between the solvent and the chains are not included, and these inertial forces are neglected. When the water is subtracted from the polymeric solution using the loss modulus from the GSER, the probe inertia is securely removed, but it is not clear if that happens with other high-frequency effects due to the solvent. The observed match at *c*/c* > 4 between the exponents obtained from *G*_*p*GSER_″ and α = 2/3 from the Zimm theory is probably related to the similarity between the subjacent models of polymer suspension, with incomplete hydrodynamic interactions, assumed for both cases.

On the other hand, the IGSER expression includes inertial effects for both bead and medium, but at least for the values of *c*/c* studied here, the IGSER by itself does not reach the 2/3 exponent, and it seems to saturate to a nearby value of α ≃ 0.7. By calculating *G*_*p*IGSER_″, we remove the Boussinesq-Basset friction force from the solvent and also from the polymer suspension before the subtraction, providing a slightly inferior exponent value, α ≃ 0.64. Therefore, both IGSER calculations seem to show inconsistencies with the assumptions of Zimm theory: *G*_I_″ by underestimating the effects of the solvent and *G*_*p*IGSER_″ by including interactions beyond the Zimm model.

### Living Polymers (WLM) Solutions

The worm-like micelles are cylindrical structures of molecules with hydrophilic and hydrophobic parts, which are spontaneously assembled in aqueous solutions. For generating these structures, we employ a well-known CPyCl/NaSal system, where cetylpyridinium chloride acts as a surfactant and sodium salicylate is the strongly binding counterion. Living polymers are expected to entangle when they overlap, unlike regular polymers, which need a concentration sufficiently higher than c* to generate this effect. The viscosity of the solution quickly increases because of the rapid growth of the worm-like structures when *c* ≥ c*, which will produce a strong viscoelastic fluid for high enough values of *c*/c*.^74^ Besides the ratio *c*/c*, these micelle structures, formed by amphiphilic molecules, are expected to be notably influenced by the water solvent, especially at lower polymer concentrations, where the entanglement of the network is weaker.

In Figure 4, we plot an analogous figure to that in Figure 2 for PEO solutions by using three representative figures of the performed experiments (see the Supporting Information document for the complete set of figures). We plot the same quantities as in Figure 2, i.e., *G*_GSER_″, its inertia-corrected version, *G*_I_″; and when subtracting water measurements, *G*_*p*_″, by using the GSER or the IGSER. The panels (a) and (b) plot the micelle suspension with polymer concentration *c* = 2 wt %, which is six times the overlap concentration, using different probe particle sizes, *a* = 0.94 μm and *a* = 1.47 μm. The experiments with increased sphere size are expected to amplify the response to inertial effects because of the influence of the solvent.^75^ This is because the Brownian probe disturbs the motion of the molecules around it, generating a velocity field that grows vorticity diffusion. When the fluid is incompressible, a backflow appears at short times, creating a vortex ring^76^ which carries the momentum in a time scale τ_*f*_ = *a* ^2^ρ/η. For a micrometer-sized probe in water, τ_*f*_ ∼ 1 μs, and increasing the sphere size for the same kind of fluid increases the characteristic time where this effect can have an influence.

**Figure 4:**
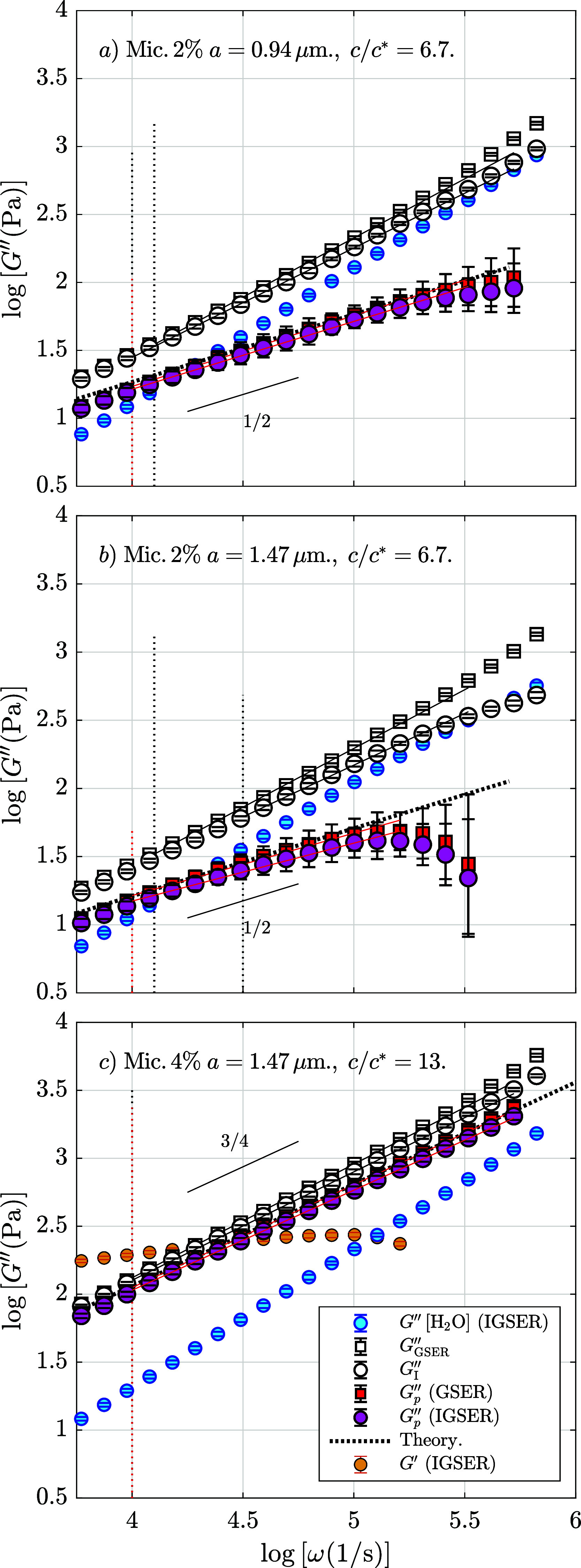
Optically trapped single-particle microrheology in worm-like micelle solutions. The loss modulus is calculated by *G*_GSER_″ (□), *G*_IGSER_″ (○), *G*_*p*GSER_″ (■—red), and *G*_*p*IGSER_″ (●—rose). We plot the water measurements (●—blue), the curves from theoretical models (.....), and linear regressions (—) where the vertical dotted lines indicate their lower boundaries. The data have been blocked in 10 bins per decade. Errors are plotted but are smaller than the point size. In (a) *c* = 2 wt %, probe particle size *a* = 0.94 μm, steady-state viscosity η_0_ = 73 mPa s, molecular mass value (free parameter, see text) *M*_w_ = 7.7 × 10^4^ kDa; (b) *c* = 2 wt %, *a* = 1.47 μm, η_0_ = 73 mPa s, *M*_w_ = 10^5^ kDa; (c) *c* = 4 wt %, *a* = 1.47 μm, η_0_ = 90 mPa s, and the storage modulus *G*′ (●—orange).

First of all, Figure 4a,b (different size particle) shows that the water loss modulus (blue data) is near to the loss modulus of the micelle solutions when using a concentration at 2 wt %, something which does not occur if the concentration is doubled, as plotted in Figure 4c, indicating a notable increase in the viscosity at that last concentration value. For 2 wt % concentration, the micelles overlap, but the entangled network is not completely formed, and at high frequencies, the loss modulus is practically the same as water. Therefore, we truncate the calculation of the water-subtracted modulus, *G*_*p*_″, at frequencies above 10^5^ Hz, depending on the probe particle size. In Figure 4b, we increase the effect of the solvent by using a bigger bead, producing a lower value of the top limit frequency. In fact, *G*_I_″ and *G*_*p*_″ deviate from the analogous curves plotted in Figure 4a since both quantities move downward when increasing the particle size. Then, in the first two panels (*c* = 2 wt %), before the observed limit frequencies, the *G*_*p*_″ curves are compatible with a theoretical Rouse behavior (dotted curves) and α = 1/2. The complex modulus for the Rouse model is obtained by eq 2, using the proper τ_*p*_ values defined by eq 3. The main issue when drawing these equations according to the experimental data is the nonfixed value of the molecular weight, *M*, because the molecular weight distribution is not constant. Here, the value of *M* is considered as a free parameter to fit the Rouse model to the experimental data (the specific obtained values are indicated in the caption of Figure 4 and in the Supporting Information document).

Regarding Figure 4c, the micelle solution (*c* = 4 wt %) shows the expected power-law behavior at high frequencies, with an exponent approximately equal to 3/4, indicating the presence of semiflexible structures of a completely formed entangled network. Here, the theoretical curve corresponds to eq 4, where the quantities which appear in the expression can be evaluated using the standard theory of polymers.^5^ Particularly (for further details of the methodology, see ref 35), the area density is ρ_m_ = ϕ/[(π/4)*d*_mic_^2^] = 8.2 × 10^15^ m^–2^, where ϕ is the volumetric concentration^77^ and the micelle diameter is *d*_mic_ = 2.5 nm.^78^ The bending modulus is κ = *k*_B_ *Tl*_p_, where *k*_B_ is the Boltzmann constant and $l_{p}={\left(k_{B}T/8\eta_{s}\omega_{0}\right)}^{1/3}$ is the persistence length. In this last quantity, ω_0_ is the frequency at which *G*′ and *G*″ cross. In Figure 4c, we plot the *G*′ values (orange points) where we can directly see that ω_0_ = 1.8 × 10^4^ s^–1^. Finally, we need to evaluate the lateral drag coefficient by ζ = 4πη_s_/ln­(*A*ξ/*a*), where *A* depends on the geometry of the polymer (usually *A* = 0.6). Here, the mesh size is $\xi ={\left(k_{B}T/G_{0}\right)}^{1/3}$ , where *G*_0_ is the elastic modulus value at the local minimum of the loss modulus, which appears at lower frequencies, for the data of Figure 4c, we have *G*_0_ = 131 Pa. A closer look into Figure 4c shows that this theoretical fitting matches better with the curves where the loss modulus of water has been subtracted (*G*_*p*_″) than with the inertia-corrected expression (*G*_I_″).

In Figure 5, we summarize the obtained α exponent values for the power-law behavior of the loss modulus in these experiments with WLM solutions. We perform measurements for three concentrations of micelles, verifying in all cases *c*/c* > 1, and two sizes of the probe particle. At first view, Figure 5 shows that removing the loss modulus of the water measurements (*G*_*p*_″, from the GSER or the IGSER) reveals a Rouse behavior (α = 1/2) at lower polymer concentrations. The other calculations for the loss modulus (*G*_I_″ and *G*_GSER_″) produce intermediate exponents, with a decreasing tendency to 3/4, similar to the transition to the 2/3 exponent for classic polymers shown in Figure 3. At *c* = 4 wt %, all calculations for the loss modulus, including *G*_*p*_″, are nearby the expected semiflexible solution behavior, with exponent α = 3/4.

**Figure 5:**
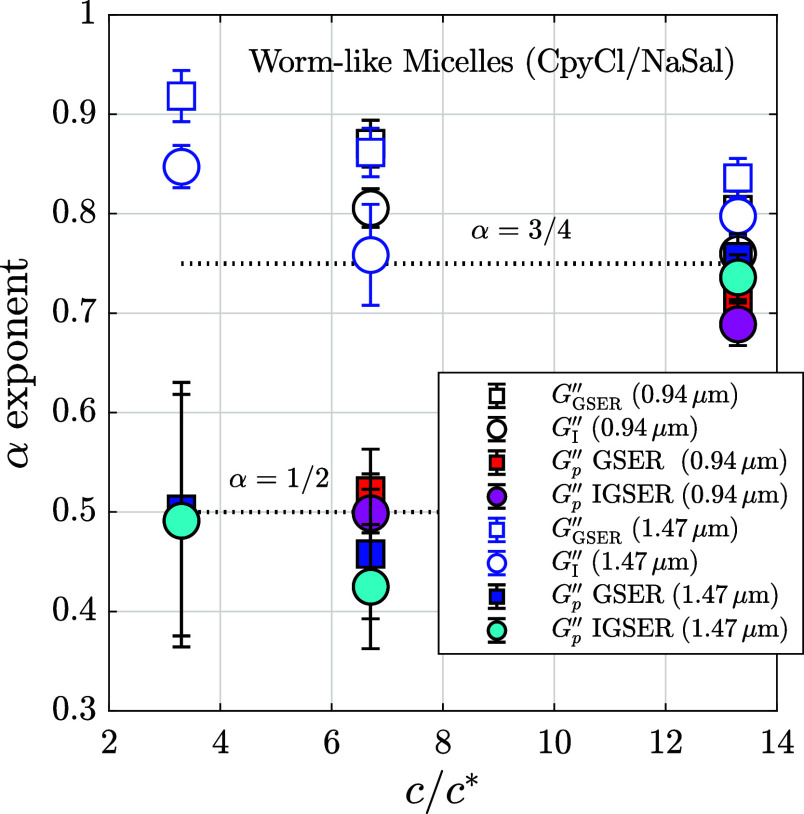
Experimental power-law exponents α in the high-frequency behavior of the loss modulus of worm-like micelle solutions as a function of *c*/c*, where *c* is the polymer concentration and c* the overlap concentration (c* = 0.3 wt %). The values of α are obtained from linear regressions calculated from *G*_GSER_″ (*a* = 0.94 μm □, *a* = 1.47 μm (blue□)), *G*_I_″ (*a* = 0.94 μm ○, *a* = 1.47 μm (blue○)), *G*_*p*GSER_″ (*a* = 0.94 μm ■—red, *a* = 1.47 μm ■—blue), and *G*_*p*IGSER_″ (*a* = 0.94 μm ●—rose, *a* = 1.47 μm ●—sky blue).

Regarding the influence of the probe size, we have already detected that a larger sphere decreases the top limit frequency when subtracting the solvent at 2% concentration, which indicates that vortex propagation might have an effect on the high-frequency behavior of these fluids. At that concentration, the inertia-corrected curve *G*_I_″, for *a* = 1.47 μm, produces an exponent value nearer to 3/4 because of these observed changes at higher frequencies. On the other hand, a mixed dependence with the particle size is observed between α values calculated through *G*_*p*_″ curves, but this might be an effect of the increased error of the exponents because of the limited region where the linear regressions can be applied.

However, the main result observed in Figures 5 and 4 is the Rouse behavior in micelle solutions at intermediate concentrations (2 ≲ *c*/c* ≲ 10), when the contribution of the solvent to the loss modulus is subtracted. We calculated *G*_*p*_″ using the GSER and the IGSER, and analogously to the results with PEO solutions, we observed that the α values from the IGSER are slightly but systematically lower than the GSER ones. This difference might be because of the friction force having an influence at high frequencies, but the values are inside the range of the calculated error intervals, and a clear distinction cannot be assured. For these intermediate concentrations, the entangled network is not completely developed, and the observed dynamics have their origin in the single-filament regime, not in the collective network dynamics. Such a behavior has been previously observed for this kind of micelle solutions at 1% concentration, where the plateau of the elastic modulus, which reflects the collective dynamics of the micelle structures, is not completely formed.^25^ In this situation, the high-frequency behavior of the complex modulus is notably affected by the “breathing modes” and the Rouse modes.^74^ The Rouse regime and its exponent 1/2 appear at high frequencies when the hydrodynamic interactions are reduced or screened,^4^ something we have probably confirmed by the almost negligible differences between GSER and IGSER calculations for *G*″. Basically, the “breathing modes” come into view when the time scale characterizing the scission of the micelles is reduced, and the polymer motion is dominated by the length fluctuations of the reptation tube.^64^ When the time scale of the breaking processes is reduced, the relaxation is independent of the micellar chain structure or configuration, and the Rouse behavior appears when the molecular motions of part of the micellar structures are on the order of the entanglement length. Therefore, in Figure 5 we observe that the experimental methodology analyzed in this work, which consists of subtracting the solution contribution, more effectively removes the effects of hydrodynamics, allowing us to observe the Rouse modes in micelle solutions at low concentration values.

## Conclusions

The mixed interactions between solvent and solute are essential, e.g., for understanding the transport behavior of macromolecules in solutions. The transport processes characterize the polymer solutions through quantities that define the dynamics of the solute, like the diffusion and sedimentation coefficients or the friction constant. These coefficients depend on the properties of the polymer and on the solvent medium but also on the mixed interactions of polymer–polymer, polymer–solvent and solvent–solvent.^79^ To study the influence of the solvent in well-known polymeric suspensions, especially regarding hydrodynamics and inertia effects, we explore the short-time/high-frequency region of the frequency-dependent complex modulus, G*­(ω), using optical trapping interferometry. Here, we focus on the short-time power-law behavior of the loss modulus, *G*″(ω), of these viscoelastic solutions, something that characterizes the individual motion of the polymers.

Our analysis shows that the classic Rouse and Zimm models and their power-law exponents at high frequency quantitatively match the experimental results when the water contribution to the modulus is adequately subtracted. This agreement appears at low polymer entanglement for classic polymers (Zimm) and micelle suspensions (Rouse). At higher levels of entanglement, the power-law exponent values for classic and living polymers behave differently: the former tends to the Rouse model exponent because of the decrease of the solvent influence, whereas the latter behaves like a suspension of semiflexible polymers. For classic polymer (PEO) solutions and when using the GSER and the IGSER methods for calculating the complex modulus, the power-law exponent α continuously decreases from α = 1 to 0.7 in the range of a relative concentration 1 < *c*/c* < 10. However, when the water measurements in the loss modulus curves are subtracted, a value near the Zimm exponent (≈0.64–0.67, depending on the methodology) is obtained when *c*/c* ⩾ 4. Such a result indicates that inertia effects can be more efficiently removed by the direct subtraction of the contribution of the solvent and that the solvent dramatically affects the detection of the entangled structures present in the polymeric solution for much lower values of the ratio *c*/c* than was previously expected. Living polymers (WLMs) generate much more complicated dynamics than classic ones, but, in a similar manner, the Rouse power-law value (≈0.5) appears in the experimental results at *c*/c* < 10 when removing the water contribution. In our experiments, the semiflexible behavior in these viscoelastic suspensions is reached at higher values of polymer concentration. Here, the change in the exponent from Rouse–Zimm to 3/4 is expected to occur at a critical frequency ω_0_ = *k*_B_ *T*/8η_s_ *l*_p_^3^, value that corresponds to the shortest relaxation time in the Rouse–Zimm spectrum.^78^ If *l*_p_ ∼ 30–40 nm at higher concentrations, this frequency is ω_0_ ∼ 10^4^ Hz, which is the lowest value of the fitting region used in this work. For lower concentration values, the persistence length is not much larger than the monomer size (*l*_*p*_ ∼ 10 nm for *c* = 1 wt %, and *d* ∼ 3 nm), and then the Rouse dynamics with 1/2 exponent at higher frequencies appear because of the reduced hydrodynamic interactions,^25^ as we have observed in this work by subtracting the water contribution.

Our study shows that, in the semidilute regime, there is a transition in the short-time power-law exponent values obtained through the complex modulus. The different regimes detected depend on the ratio of the polymer concentration to the overlap concentration, i.e., on the degree of network interconnection, and especially they depend on the influence of the mixed interactions between the solvent and the solute. While the Zimm model has been successful at reproducing dilute polymer solutions in θ-conditions, and the Rouse model works particularly well with melted suspensions, the Rouse–Zimm model has been demonstrated to be incomplete for reproducing the generic dynamical behavior of polymers in dilute solutions.^4^ Our results confirm that these classic models, without modifications or generalizations, work properly in semidilute viscoelastic suspensions when the influence of the solvent is subtracted because the Rouse–Zimm theory does not include full hydrodynamic effects and the inertia of the fluid and the bead when the polymer is moving in the solvent has to be included to correctly reproduce the high-frequency behavior. Our experimental work indicates that the influence of the solvent in semidilute polymeric suspensions is not well understood at present and that further investigations, both experimental and theoretical, should be developed.

## Supplementary Material



## Notes

Experimental methodology details, table of results, and figures not included in the main manuscript (PDF)
