# Supplementary material for: Rouse and Zimm Short-Time Exponents When Subtracting the Solvent Contribution in Semidilute Polymeric Solutions
Source: Macromolecules. 2025 Jul 23;58(15):8107–17. doi: 10.1021/acs.macromol.5c00371 (PMC12356060; doi:10.1021/acs.macromol.5c00371)
Supplement: Supplementary file 1 [file ma5c00371_si_001.pdf]

Supplementary Information for: Rouse and Zimm short-time exponents when subtracting the solvent contribution in semidilute polymeric solutions.

Pablo Domínguez-García
Dep. Física Interdisciplinar, Universidad Nacional de Educación a Distancia (UNED), Madrid, Spain

Sylvia Jeney
Department of Physics, University of Fribourg, Fribourg, Switzerland

In this supplemental document we include some aspects of the experimental methods not detailed in the main paper, the whole set of curves which have not been added in the main manuscript, and a table which summarizes all the power-law exponents calculated for each of the solutions studied here.

I. EXPERIMENTAL METHODOLOGY

To initially analyze the bead motion, we use the mean square displacement (MSD), which is obtained in linear scale because the experimental set-up measures the motion of the optically trapped probe in fixed time-steps. A linear plot will generate an excess of experimental points on the left part of the curves. To avoid this effect and for the calculation of the power-law exponents, the data plots need a double-axis logarithmic scale and a logarithmic blocking of the calculated data.

The blocking method divides the abscissa of the plot in blocks which are equally distributed intervals in logarithmic scale. All the data points inside the block are averaged and their errors calculated [1, 2]. We use ten bins per decade for assuring a good visualization of the experimental data. However, at short-time scales $\sim 1\,\mu$s, the equally spaced blocked points are not generated, because the lag time is $\Delta t = 1\,\mu$s. For this reason, we restricted the analysis to larger time, i.e., a frequency of $\sim 10^{5.7}$ Hz.

Here, the values of the power-law exponents are calculated by linear regressions at logarithmic scale to the blocked data in the loss modulus. For this calculation, we need to define criteria for the bottom and top limits. The top frequency has been already defined (exponent 5.7), and the bottom is considered to be an 10^4 Hz for theoretical reasons (see main text). However, to assure that we are calculating only one power-law behavior, the following procedure is applied: first, we perform a linear regression using the last five points at the larger frequencies and we calculate the residual of the last frequency (5.7), i.e., the difference between the value calculated by the linear regression at this frequency and the experimental one. Then, if the relative error is inferior to a value of reference (2% for PEO solutions and 4% for micelles solutions), a new point is added to the regression. The process is repeated until reaching a relative error greater than the value of reference. In all the figures, we have plotted the lower limits of the linear regressions using dotted vertical lines.

Regarding the calculation of the complex modulus, any variation of the basic assumptions needed for that calculation will generate anomalous effects in the results when obtaining G^*, i.e., variations to a situation where the bead is moving in a continuum mechanical environment in thermal equilibrium or sufficiently close to it [3]. In the classic methodologies for calculating the complex modulus (GSER and Kramers-Kronig integrals [4]) a characteristic breakup for the elastic modulus, G', at high frequencies is observed for our experimental system [5, 6]. This behavior, which appears decades before the influence of the Nyquist frequency [7], is likely to occur because of the greater sensitivity of the cosine calculation at low values of t. The Mason's approximation used in the GSER calculation of the complex modulus uses $\alpha(s) \equiv (d \ln \mathrm{MSD}(t)/ \ln t)|_{t=1/s}$, to locally expand the MSD around the frequency of interest s. If $\alpha \sim 1$ over a large temporal range, the estimate for the dominant $G''(\omega)$ is excellent, but $G'(\omega)$ degrades. Besides, the elastic component tends to be very sensitive to artifacts [8, 9]. Therefore, the calculations for the elastic modulus, $G'(\omega)$ in this system should be limited at ~ 10 kHz, so the power-law behavior has to be studied through the loss modulus, $G''(\omega)$.

II. RESULTS

In this section, we add all data and figures not included in the representative examples of the main manuscript. In Table 1S, we summarize all the obtained values for the power-law exponents for every experiment (plotted in Figs. 3 and 5) among the data which characterize the polymer aqueous solutions, while Fig. 1S includes the loss modulus curves (similar to Figs. 2 and 4) not showed in the main manuscript.

TABLE 1S: Power-law exponents (α) for all the fluids studied. We include the following data from the polymer solutions: type of polymer, molecular weight M_w (the apparent values for WLM are obtained by fitting the theoretical curves, see Fig 4 c) and Fig 1S h)), c is the polymer concentration, a is the radius of the probe, η_0 is the steady-state viscosity of the fluid, c^* is the overlap concentration, c/c^* is the ratio between polymer and overlap concentration. The α values correspond to the linear regressions in double logarithmic scale for the four curves of the loss modulus used to analyze the data.

Polymer	M_w(kDa)	$c(\frac{\text{mg}}{\text{ml}})$	$a(\mu\text{m})$	η_0(mPa.s)	c^* $(\frac{\text{mg}}{\text{ml}})$	c/c^*	$\alpha\, G''_{\text{GSER}}$	$\alpha\, G''_{\text{IGSER}}$	$\alpha\, G''_{p\,\text{GSER}}$	$\alpha\, G''_{p\,\text{IGSER}}$
PEO	231	0.5	0.94	1.0	4.3	0.1	1.04 ± 0.02	0.99 ± 0.02	1.04 ± 0.03	1.00 ± 0.03
PEO	747	0.5	0.94	1.1	1.8	0.3	1.03 ± 0.03	0.97 ± 0.02	1.00 ± 0.04	0.94 ± 0.04
PEO	231	4	0.94	1.8	4.3	0.9	0.98 ± 0.02	0.93 ± 0.02	0.91 ± 0.03	0.88 ± 0.03
PEO	495	2.5	0.94	4.3	2.4	1.0	0.99 ± 0.03	0.92 ± 0.02	0.86 ± 0.04	0.82 ± 0.04
PEO	747	2.5	0.94	3.5	1.8	1.4	0.96 ± 0.03	0.90 ± 0.02	0.82 ± 0.03	0.80 ± 0.03
PEO	231	10	0.94	4.9	4.3	2.3	0.88 ± 0.02	0.84 ± 0.02	0.78 ± 0.03	0.76 ± 0.03
PEO	231	15	0.94	10	4.3	3.5	0.81 ± 0.02	0.77 ± 0.02	0.73 ± 0.02	0.70 ± 0.02
PEO	495	10	0.94	6.7	2.4	4.2	0.84 ± 0.04	0.77 ± 0.02	0.72 ± 0.02	0.69 ± 0.02
PEO	495	15	0.94	12	2.4	6.3	0.79 ± 0.04	0.70 ± 0.02	0.66 ± 0.02	0.63 ± 0.02
PEO	747	15	0.94	68	1.8	8.3	0.77 ± 0.04	0.70 ± 0.02	0.67 ± 0.03	0.64 ± 0.03
WLM	$7.7\,10^4$	20	0.94	73	3.0	66.7	0.87 ± 0.02	0.81 ± 0.02	0.52 ± 0.04	0.50 ± 0.04
WLM	-	40	0.94	380	3.0	133.3	0.80 ± 0.02	0.76 ± 0.02	0.72 ± 0.02	0.69 ± 0.02
WLM	$7\,10^4$	10	1.47	22	3.0	33.3	0.92 ± 0.03	0.85 ± 0.02	0.50 ± 0.13	0.49 ± 0.13
WLM	10^5	20	1.47	73	3.0	66.7	0.86 ± 0.02	0.76 ± 0.05	0.46 ± 0.07	0.43 ± 0.06
WLM	-	40	1.47	380	3.0	133.3	0.84 ± 0.02	0.80 ± 0.02	0.76 ± 0.02	0.74 ± 0.02

[1] H. Flyvbjerg and H. G. Petersen, J. Chem. Phys. **91**, 461 (1989).

[2] A. Córdoba and J. D. Schieber, Rheol. Acta **61**, 49 (2022).

[3] E. M. Furst and T. M. Squires, *Microrheology* (OUP Oxford, 2017).

[4] P. Domínguez-García, G. Dietler, L. Forró, and S. Jeney, Soft Matter **20**, 4234 (2020).

[5] P. Domínguez-García, F. Cardinaux, E. Bertseva, L. Forró, F. Scheffold, and S. Jeney, Phys. Rev. E **90**, 060301 (2014).

[6] P. Domínguez-García, J. R. Pinto, A. Akrap, and S. Jeney, Appl. Phys. Lett. **125**, 173702 (2024).

[7] K. Nishi, M. L. Kilfoil, C. F. Schmidt, and F. C. MacKintosh, Soft Matter **14**, 3716 (2018).

[8] T. Savin and P. S. Doyle, Phys. Rev. E **76**, 021501 (2007).

[9] P. Domínguez-García and M. A. Rubio, Appl. Phys. Lett. **102**, 074101 (2013).

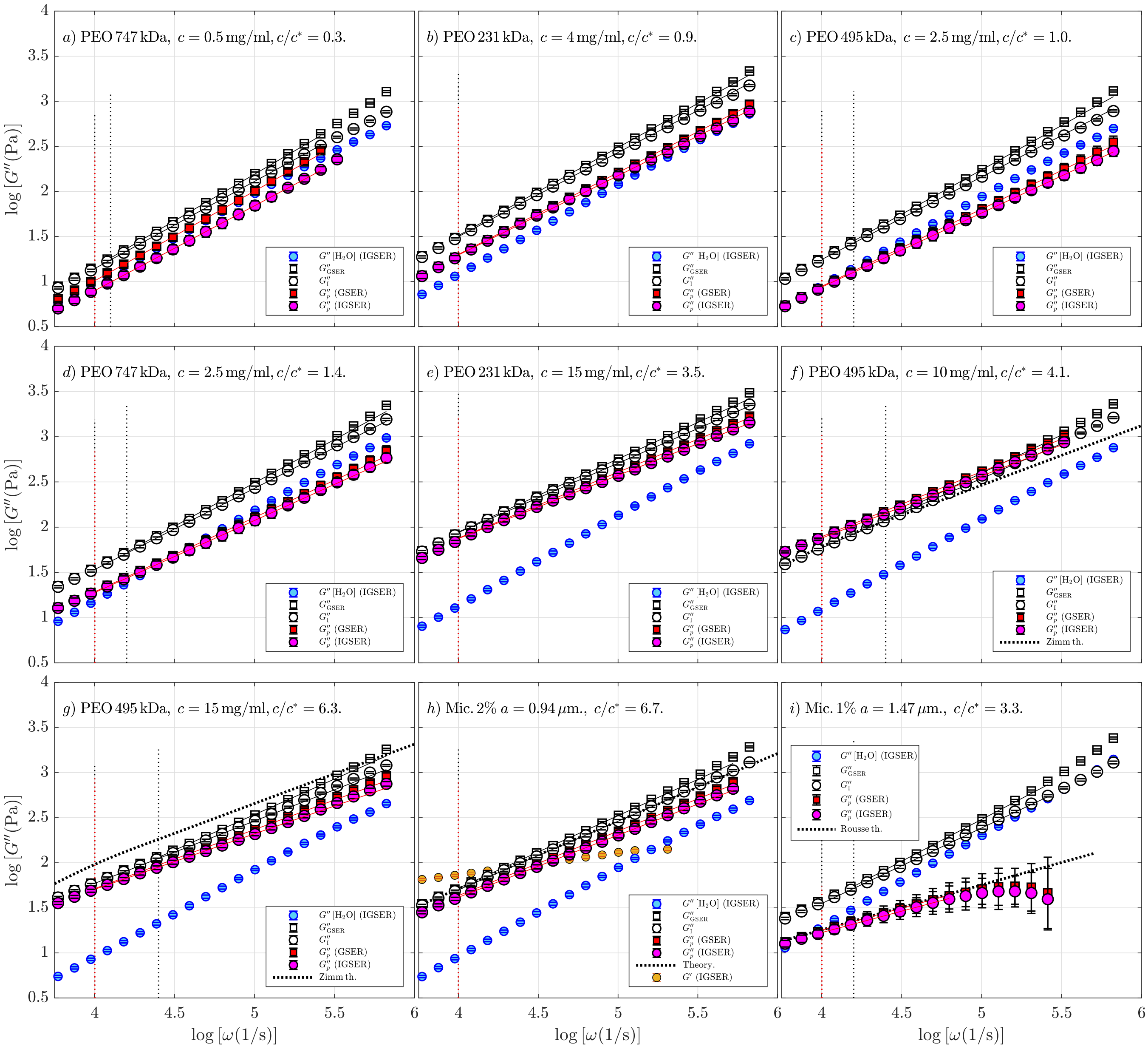

FIG. 1S:) Loss modulus, $G''(\omega)$, obtained by optically-trapped single-particle microrheology for poly(ethylene) oxide (PEO) solutions (from a) to g)) and worm-like micelle (WLM) solutions (h) and i). The data have been blocked in 10 bins per decade. Errors are plotted, but are smaller than the points size. The loss modulus is calculated by different methods: G''_{GSER} (□); its inertia-corrected version G''_{I} (○); and when subtracting water measurements from GSER calculations $G''_{p\,\mathrm{GSER}} = G''_{\mathrm{GSER}} - G''_{\mathrm{GSER}}(\mathrm{H_2O})$ (■), and from IGSER calculations $G''_{p\,\mathrm{IGSER}} = G''_{\mathrm{IGSER}} - G''_{\mathrm{IGSER}}(\mathrm{H_2O})$ (●). We plot the water measurements from IGSER (●), the curve from Zimm theory (······), and linear regressions (——), where the vertical dotted lines indicate their lower boundaries. We plot the storage modulus $G'(\mathrm{IGSER})$ (●) in h) for the case where the 3/4 power-law exponent is found. The ratios c/c^* values are indicated inside the plots, the corresponding steady-state viscosities η_0, and the fitted M_w values for WLM solutions can be consulted in Table 1S.
